# Time-dependent parameter of perfusion imaging as independent predictor of clinical outcome in symptomatic carotid artery stenosis

**DOI:** 10.1186/s12883-016-0576-5

**Published:** 2016-04-19

**Authors:** Sibu Mundiyanapurath, Peter Arthur Ringleb, Sascha Diatschuk, Oliver Eidel, Sina Burth, Ralf Floca, Markus Möhlenbruch, Wolfgang Wick, Martin Bendszus, Alexander Radbruch

**Affiliations:** Department of Neurology, University Hospital Heidelberg, Im Neuenheimer Feld 400, Heidelberg, 69120 Germany; Department of Neuroradiology, University Hospital Heidelberg, Im Neuenheimer Feld 400, Heidelberg, 69120 Germany; German Cancer Research Center, Department of Radiology, INF 280, Heidelberg, 69120 Germany; CCU Neurooncology, German cancer Consortium (DKTK) & German Cancer Research Center (DKFZ), INF 280, Heidelberg, 69120 Germany

**Keywords:** Ischemic stroke, Magnetic resonance perfusion, Carotid stenosis, Tmax, TTP

## Abstract

**Background:**

Carotid artery stenosis is a frequent cause of ischemic stroke. While any degree of stenosis can cause embolic stroke, a higher degree of stenosis can also cause hemodynamic infarction. The hemodynamic effect of a stenosis can be assessed via perfusion weighted MRI (PWI). Our aim was to investigate the ability of PWI-derived parameters such as TTP (time-to-peak) and T_max_ (time to the peak of the residue curve) to predict outcome in patients with unilateral acute symptomatic internal carotid artery (sICA) stenosis.

**Methods:**

Patients with unilateral acute sICA stenosis (≥50 % according to NASCET), without intracranial stenosis or occlusion, who underwent PWI, were included. Clinical characteristics, volume of restricted diffusion, volume of prolonged TTP and T_max_ were retrospectively analyzed and correlated with outcome represented by the modified Rankin Scale (mRS) score at discharge. TTP and T_max_ volumes were dichotomized using a ROC curve analysis. Multivariate analysis was performed to determine which PWI-parameter was an independent predictor of outcome.

**Results:**

Thirty-two patients were included. Degree of stenosis, volume of visually assessed TTP and volume of TTP ≥2 s did not distinguish patients with favorable (mRS 0–2) and unfavorable (mRS 3–6) outcome. In contrast, patients with unfavorable outcome had higher volumes of TTP ≥4 s (9.12 vs. 0.87 ml; *p* = 0.043), TTP ≥6 s (6.70 vs. 0.20 ml; *p* = 0.017), T_max_ ≥4 s (25.27 vs. 0.00 ml; *p* = 0.043), T_max_ ≥6 s (9.21 vs. 0.00 ml; *p* = 0.017), T_max_ ≥8 s (6.86 vs. 0.00 ml; *p* = 0.011) and T_max_ ≥10s (5.94 vs. 0.00 ml; *p* = 0.025) in univariate analysis. Multivariate logistic regression showed that NIHSS score on admission (Odds Ratio (OR) 0.466, confidence interval (CI) [0.224;0.971], *p* = 0.041), T_max_ ≥8 s (OR 0.025, CI [0.001;0.898] *p* = 0.043) and TTP ≥6 s (OR 0.025, CI [0.001;0.898] *p* = 0.043) were independent predictors of clinical outcome.

**Conclusion:**

As they stood out in multivariate regression and are objective and reproducible parameters, PWI-derived volumes of T_max_ ≥8 s and TTP ≥6 s might be superior to degree of stenosis and visually assessed TTP maps in predicting short term patient outcome. Future studies should assess if perfusion weighted imaging might guide the selection of patients for recanalization procedures.

**Electronic supplementary material:**

The online version of this article (doi:10.1186/s12883-016-0576-5) contains supplementary material, which is available to authorized users.

## Background

Internal carotid artery stenosis causes about 10–15 % of ischemic strokes which remain a leading cause for disability [1]. Generally, the degree of stenosis is defined by the North American Symptomatic Carotid Endarterectomy Trial (NASCET) differentiating 10 to 90 % stenosis based on the diameter distal to the stenosis in relation to the diameter in the stenosis itself [2]. Based on current guidelines, treatment with thromboendarterectomy (TEA) or carotid artery stenting (CAS) is indicated in patients with a symptomatic stenosis of greater than 50 % [2, 3]. A hemodynamically relevant stenosis usually has a degree of at least 70 % with a decrease of the poststenotic peak systolic velocity, increased pulsatility of the prestenotic common carotid artery and a decrease of the slope to peak systolic velocity in the transcranial Doppler sonography [4]. This type of stenosis can cause, in addition to embolic strokes, hemodynamic strokes of the watershed areas [5]. The degree of hemodynamic impairment can be assessed on a capillary level using magnetic resonance perfusion weighted imaging (PWI). In contrast to the measurement of the degree of the stenosis, this method takes all compensatory mechanisms - especially collateral blood flow - into account. It is known that carotid artery stenosis changes the time to the maximum of the tissue concentration time curve (time-to-peak – TTP) in PWI [6]. However, it is not known if these changes in TTP correlate with outcome. It was rather reported that the use of TTP maps may result in an overestimation of the tissue at risk [7]. In addition, visually assessed TTP maps are still frequently used in clinical routine although TTP maps with thresholds seem to be superior [8]. An alternative PWI parameter that has been used more frequently within recent years is T_max_, which is the time to the maximum of the residual curve (obtained by deconvolution of the tissue concentration time curve) [9–11]. Even though it seems obvious that T_max_ or TTP are superior to the degree of stenosis in depicting clinically relevant hypoperfusion in patients with carotid artery stenosis, it has not been demonstrated in detail yet and patients with higher degree of stenosis are commonly suspected to have higher degrees of hypoperfusion.

In the current study, we analyzed TTP and T_max_ maps and their ability to predict short term outcome in patients with unilateral acute symptomatic internal carotid artery (sICA) stenosis.

## Methods

### Patient selection

Patients with symptomatic (stroke or transient ischemic attack) unilateral carotid artery stenosis ≥50 % (according to NASCET) without high grade intracranial stenosis or occlusion (in Doppler sonography or angiography) who were examined with MRI including PWI were selected from the hospital database. The carotid artery stenosis had to be the most likely etiology of the stroke/transient ischemic attack, determined by an experienced vascular neurologist blinded to this analysis. Age, side of stenosis, degree of stenosis (according to NASCET), additional therapy (thrombolysis, TEA, CAS), risk factors (atrial fibrillation, peripheral artery disease, smoking, hypercholesterolemia, hypertension, diabetes), National Institute of Health Stroke Scale (NIHSS) score on admission and on discharge, modified Rankin (mRS) score on admission and on discharge were recorded.

Clinical outcome was assessed at discharge by an investigator not blinded to the treatment but to this analysis by clinical examination using the mRS. Favorable outcome was defined as a score of 0 to 2 on the mRS, reflecting the ability to live independently. This definition is commonly used in stroke trials [12]. Unlike the large stroke trials, four patients with a premorbid mRS of three were included in our study. For these patients an unchanged mRS at discharge was defined as favorable outcome. One patient had a premorbid mRS of four and a mRS at discharge of five and was therefore defined to have an unfavorable outcome.

### Image acquisition

Images were acquired during routine clinical diagnostics using a three Tesla MR system (Magnetom Tim Trio or Verio with identical technical parameters, Siemens Healthcare, Erlangen, Germany) with a 12-channel head-matrix coil. For dynamic susceptibility contrast perfusion imaging, 0.1 mmol/kg gadolinium based contrast medium (Dotarem®, Guerbet) was administered and images were obtained with a GRE echo planar imaging (EPI) sequence: TE 35 ms, TR 1920 ms, FoV 240 × 240 mm, slice thickness 5 mm, 75 dynamic scans (0.1 mmol/kg Dotarem® 3.5 ml/s using a power injector, injection after the third frame), resulting in an acquisition time of 2:31 min. TTP maps for visual assessment were calculated using the software supplied by the manufacturer (Syngo Software, Leonardo, Siemens Medical Systems, Erlangen, Germany). DWI was performed using a single-shot spin-echo (SE) echo-planar sequence with the following parameters: echo time (TE) = 90 ms, repetition time (TR) = 5300 ms, flip angle (FA) = 90°, slice thickness = 5 mm. Diffusion sensitizing gradients were applied sequentially in the x, y, and z directions with b-values of 0 and 1200 s/mm^2^. ADC trace maps were created automatically using the above mentioned software, supplied by the manufacturer.

### Image analysis

Analysis of visually assessed TTP was done using an open-source segmentation-software (ITK-SNAP, www.itksnap.org) [13]. All areas presenting an increase of TTP compared to the contralateral side on the maps provided by the scanner software (visually assessed TTP) were manually delineated on all affected slides. Subsequently, the volume of all areas was calculated.

In contrast, TTP maps with thresholds and T_max_ maps with thresholds were calculated automatically using the Olea-Sphere® software (Olea Medical®, La Ciotat, France). Whole brain automatic detection for the arterial input function [14] and block-circulant matrix without minimization of oscillation single value decomposition deconvolution (cSVD, truncation threshold 0.1) were used. No model fitting for smoothing was applied. Motion correction was achieved using an algorithm with pairwise in-plane rigid co-registration of all raw images of a given slice with a well-chosen reference image over time. It is based on minimizing a robust and computationally friendly distance between this reference image and the target image. In order to avoid local minima, a quick, coarse grain registration algorithm based on geometric information is used to initialize the fine grain minimization algorithm. Normal white matter from the unaffected middle cerebral artery territory (the M5 area from the Alberta Stroke Program Early CT Score) contralateral to the stenosis was chosen for normalization of the TTP values derived from Olea-Sphere manually using ITK-SNAP. This allowed pooling of patient data [15]. Normalization is not required for T_max_ values. The maps were grouped by values and the respective volumes were computed using in-house software created with MATLAB (MathWorks®, Natick, MA, USA) by one of the authors (SD). For TTP, the groups were: ≥2, ≥4 and ≥6 s. For T_max_, the groups were: ≥4, ≥6, ≥8 and ≥10 s. We chose to include the visual assessment of TTP maps and the automated approach because the visual assessment used to be the standard procedure in daily patient care at our hospital at the time the study was conducted and we were eager to know whether there were substantial differences to the maps calculated by a perfusion software. All images were manually checked and corrected for artifacts using ITK-SNAP. As no brain extraction software was used, artifacts in ventricles and larger subarachnoid spaces had to be removed manually. It is not possible to identify structures as the ventricles precisely on TTP and T_max_ maps. Therefore, PWI images were aligned with T2-images to facilitate artifact detection using an FSL-based algorithm (FLIRT, linear rigid registration using normalized mutual information with 6° of freedom). DWI lesions were segmented automatically using a previously published algorithm [16]. Image reading was done blinded to outcome parameters.

### Doppler sonography

The degree of the stenosis was determined by routine continuous-wave Doppler sonography using a 4 MHz extracranial Doppler probe (SONARA system, medilab®, Estenfeld, Germany) and a linear 5–10 Mhz duplex probe with color Doppler-assisted imaging according to the NASCET criteria based on criteria defined by the German Society of Sonography in Medicine (DEGUM) [4].

Transcranial Doppler sonography (TCD) was performed in routine clinical workup with a 2 Mhz pulse-wave probe using the SONARA system. The frequencies used in the study were collected by probing the middle cerebral artery at 50 mm depth. The extent of flow distraction – reduced acceleration time – in the middle cerebral artery (MCA) was assessed based on acoustic impression compared to the normal contralateral side.

Ultrasound was performed by a neurologist or a technician with extensive experience.

### Statistical analysis

Statistical analysis was conducted using Microsoft Excel® Version 2010 and IBM SPSS® Version 21. Correlation analysis was carried out using Pearson correlation coefficient. ROC-curve analysis was performed including thresholds in case of positive classification and assuming a non-parametric distribution of the area under the curve. The cut-off volume for TTP and T_max_ for the multivariate analysis was chosen from the ROC-curve, being the closest point to the upper left corner. Pretesting for normal distribution was not performed, as a two stage procedure with preliminary testing for normal distribution causes an increase of the conditional type 1 error rate [17]. Hence, univariate analysis was performed using Mann-Whitney-U and Chi-square/Fisher exact test depending on the level of measurement and the size of the tested groups. An α-Level of 0.05 was chosen. Multivariate analysis was done applying likelihood-ratio test based forward selection within binary logistic regression models where variables were included if the related *p*-value was above 0.05 and removed if the related *p*-value was above 0.10. Two-sided *p*-values are reported throughout.

## Results

**Fig. 1:**
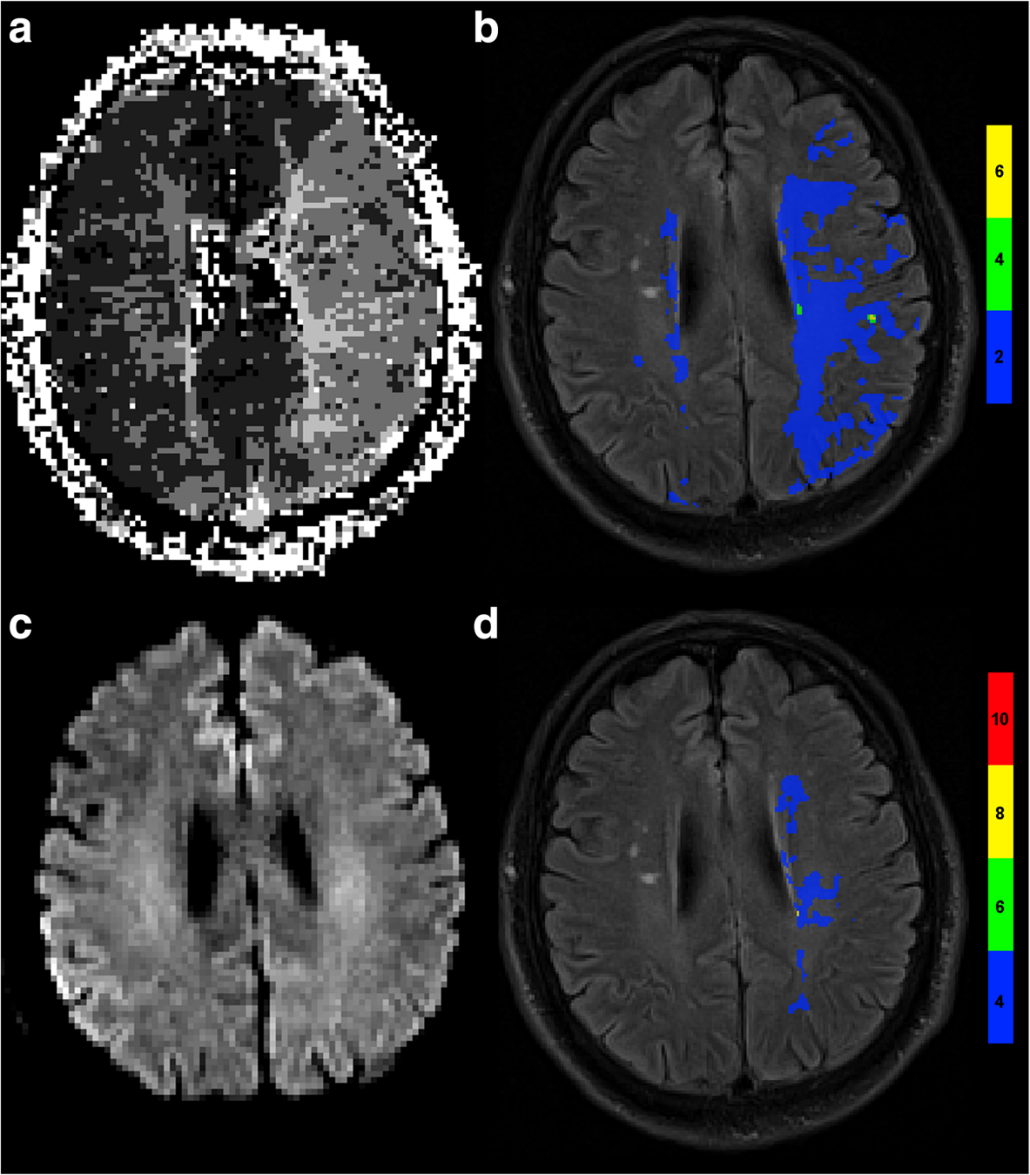
Patient with 90 % carotid artery stenosis and favorable outcome. **a** Large prolonged TTP volume on TTP-map provided by scanner software without thresholding. **b** Slight prolongation of TTP volume on TTP-map calculated by Olea-Sphere®. Maps were visualized on fluid attenuated weighted images (FLAIR). **c** DWI provided by scanner software showing no restrictions. **d** Small prolonged T_max_ volume calculated by Olea-Sphere®

**Fig. 2:**
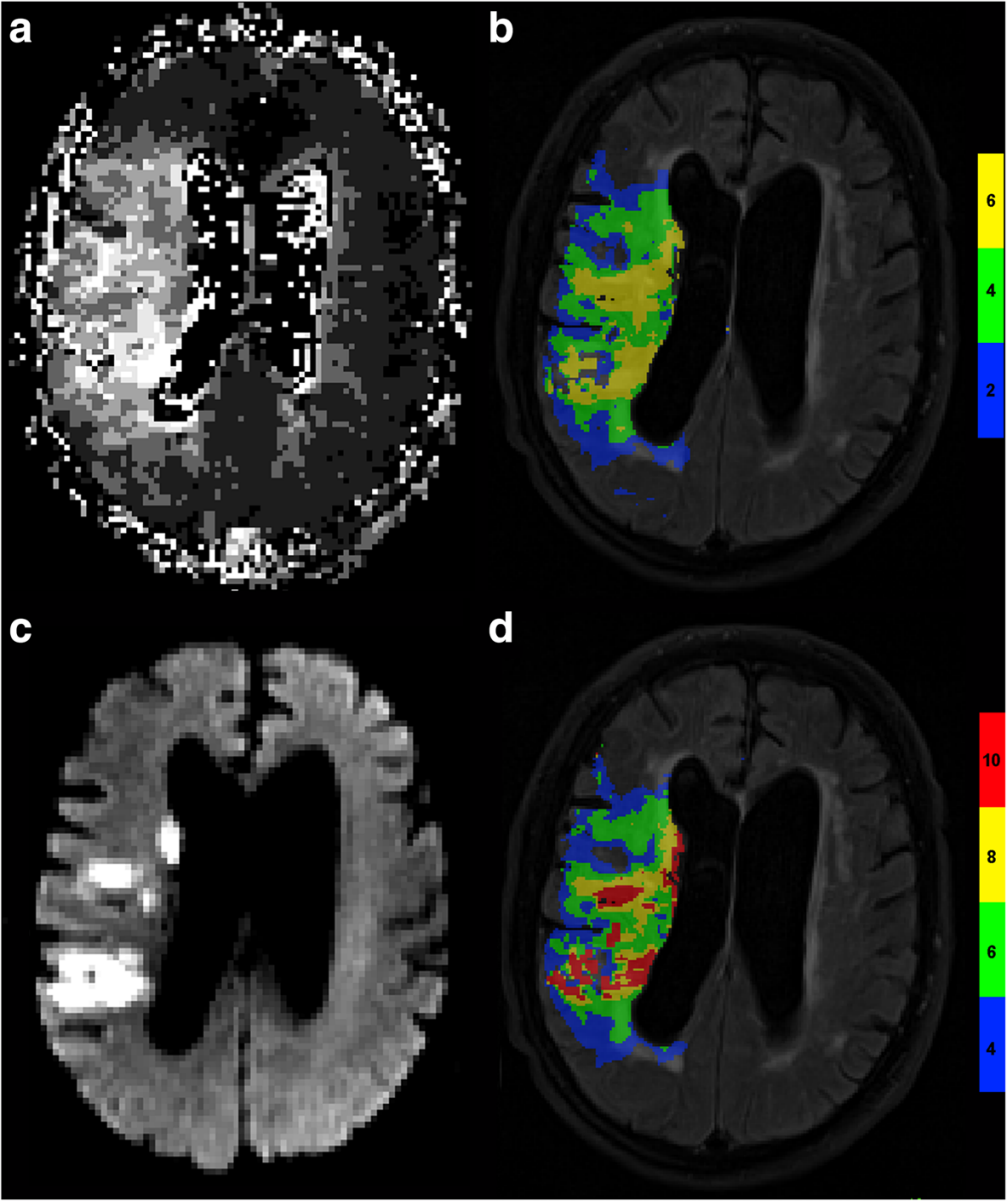
Patient with 90 % carotid artery stenosis and unfavorable outcome. **a** Large prolonged TTP volume on TTP-map provided by scanner software without thresholding. **b** Large TTP volume with markedly prolonged TTP on TTP-map calculated by Olea-Sphere®. **c** DWI provided by scanner software showing multiple areas with restricted diffusion. High grade intracranial stenosis or occlusion was excluded in angiography. **d** Large prolonged T_max_ volume calculated by Olea-Sphere®

Thirty-two patients were eligible for analysis. All patients suffered from acute sICA stenosis (ischemic stroke or transient ischemic attack) and were treated on our stroke unit. The patients had the following degrees of stenosis: 90 % (*n* = 9), 80 % (*n* = 9), 70 % (*n* = 8), 60 % (*n* = 1), 50 % (*n* = 5). None of the patients died and 23 had a favorable outcome. The median length of stay in hospital was 6.5 days (interquartile range (IQR): 4.3;8.8). Among patients with high grade stenosis and large prolonged visually assessed TTP volume, some had a small pathological perfusion volume on T_max_ maps, only a slight prolongation of the TTP in the TTP maps with thresholds (≥2 s) and favorable outcome (Fig. 1). On the other hand, some of these patients had a large pathological perfusion volume with higher values of TTP/T_max_ and unfavorable outcome (Fig. 2). Additional middle cerebral artery stenosis or occlusion was ruled out by CT angiography for this patient (Additional file 1: Figure S1). We therefore investigated whether and at what threshold pathological TTP and T_max_ volume can predict outcome using a ROC analysis. The visually assessed TTP volume and the volume with a TTP ≥2 s failed to differentiate between favorable and unfavorable outcome (AUC = 0.560; *p* = 0.600 and AUC = 0.686; *p* = 0.107, respectively). All other PWI-derived parameters were significantly different in patients with favorable and unfavorable outcome in the ROC-analysis (Additional file 2: Table S1) and in univariate analysis that included other clinical parameters (age, side and degree of stenosis, risk factors, treatment, time from symptom onset to MRI, NIHSS score, mRS score and TCD parameters) as well (Table. 1). For additional multivariate analysis, a threshold for T_max_ and TTP maps was chosen from the ROC curve, which resulted in a sensitivity of 0.778 for all parameters (Additional file 2: Table S1). The T_max_ and TTP parameter with the highest area under the curve (T_max_ ≥8 s and TTP ≥6 s) were used in multivariate logistic regression analysis that included all significant (*p* <0.05) parameters from the univariate analysis as well. As a significant collinearity of the TTP and T_max_ parameter was evident, we performed two separate regression analyses using only one of the perfusion parameters in each of them. The dichotomized volume of T_max_ ≥8 s (threshold >0.94 ml, odds ratio 0.026; 95 % confidence interval [0.001;0.925]; *p* = 0.045) and TTP ≥6 s (threshold 1.42 ml, odds ratio 0.026; 95 % confidence interval [0.001;0.925]; *p* = 0.045) were independent predictors of outcome even though NIHSS score on admission and DWI lesion volume were included (Table 2).

**Table 1:** Univariate Analysis comparing patients with favorable (mRS 0–2 or unchanged) and unfavorable outcome. Displayed as median with interquartile range in parentheses or percentage

	Favorable outcome (*n* = 23)	Unfavorable outcome (*n* = 9)	*p*-value
Age	70 (63;74)	79 (74;84)	0.001
Men	74 %	89 %	0.357
Degree of Stenosis in %	80 (70;90)	80 (70;90)	0.536
Risk Factors			
-atrial fibrillation	4 %	22 %	0.119
-peripheral artery disease	17 %	11 %	0.660
-coronary heart disease	22 %	22 %	0.976
-smoking	39 %	11 %	0.124
-hypercholesterolemia	48 %	67 %	0.337
-hypertension	83 %	67 %	0.370
-diabetes	17 %	67 %	0.007
Treatment with Alteplase	9 %	11 %	0.833
Treatment with TEA or CAS	96 %	67 %	0.026
Time from symptom onset to MRI			0.884
-<24 h	57 %	78 %	
-24–48 h	13 %	11 %	
-48–72 h	4 %	0 %	
->72 h	26 %	11 %	
Time to intervention (days)	4.0 (1.5;7.0)	1.5 (0.0;3.8)	0.085
Length of stay in hospital	7 (7;9)	6 (4;11)	0.711
NIHSS score			
-on admission	3 (1;5)	8 (5;13)	0.002
-at discharge	1 (0;2)	13 (5;14)	≤0.001
Premorbid mRS Score	2 (0;2)	1 (1;2)	0.965
TCD			
-SF (kHz)	2.15 (1.65;2.40)	1.70 (1.30;2.10)	0.148
-DF (kHz)	0.75 (0.60;1.05)	0.55 (0.43;0.83)	0.122
-Mean (kHz)	1.13 (1.00;1.53)	0.93 (0.72;1.25)	0.148
-contralateral SF (kHz)	1.95 (1.70;2.78)	1.6 (1.6; 1.6)	0.634
-contralateral DF (kHz)	0.80 (0.60;0.90)	0.6 (0.3;0.6)	0.559
-mean contralateral (kHz)	1.17 (0.95;1.53)	0.93 (0.73;0.93)	0.559
-reduced acceleration at MCA	26 %	22 %	0.531
-RI	0.59 (0.56;0.67)	0.65 (0.58;0.71)	0.249
-Contralateral RI	0.66 (0.57;0.71)	0.66 (0.63;0.66)	0.712
TTP volume (ml)			
-TTP visual	78.50 (0.00;189.50)	111.40 (0.00; 232..40)	0.112
-TTP ≥2 s	18.50 (0.00;41.19)	29.77 (5.18;132.89)	0.003
-TTP ≥4 s	0.87 (0.00;4.05)	9.12 (2.19;66.86)	0.043
-TTP ≥6 s	0.20 (0.00;0.89)	6.70 (1.07;37.48)	0.017
T_max_ volume (ml)			
-T_max_ ≥4 s	0.00 (0.00;13.52)	25.27 (1.17;110.40)	0.043
-T_max_ ≥6 s	0.00 (0.00;1.10)	9.21 (0.72;44.94)	0.017
-T_max_ ≥8 s	0.00 (0.00;0.66)	6.86 (0.56;23.47)	0.011
-T_max_ ≥10s	0.00 (0.00;0.54)	5.94 (0.56; 23.47)	0.019
DWI lesion volume (ml)	2.45 (0.14;9.73)	18.04 (8.80; 24.41)	0.001

*TEA* thromboendarterectomy, *CAS* carotid artery stenting, *MRI* magnetic resonance imaging, *NIHSS* National Institute of Health Stroke Scale, *mRS* modified Rankin scale, *TCD* transcranial dopplersonography, *MCA* middle cerebral artery, *RI* resistance index, *TTP* time-to-peak, *T*_*max*_ time to maximum of residual curve

**Table 2:** Last step of multivariate logistic regression. Included parameters: NIHSS score on admission, diabetes, T_max_ ≥8 s dichotomized, TTP ≥6 s dichotomized, DWI lesion volume, treatment with TEA or CAS and age

	Odds ratio (95 % CI)	*p*-value
NIHSS score on admission	0.468 (0.224;0.975)	0.043
Diabetes	0.034 (0.001;1.639)	0.087
T_max_ ≥8 s dichotomized	0.026 (0.001;0.925)	0.045
TTP ≥6 s dichotomized	0.026 (0.001;0.925)	0.045

*NIHSS* National Institue of Health Stroke Scale, *TTP* time-to-peak, *T*_*max*_ time to maximum of residual curve

We also investigated the effect of the volume of hypoperfused tissue on another outcome parameter, NIHSS score at discharge. All perfusion parameters showed significant correlations with the NIHSS score at discharge. However, the volume of visually assessed TTP (correlation coefficient (r) = 0.36, *p* = 0.041) showed a markedly lower and less significant correlation compared to TTP ≥2 s (*r* = 0.62, *p* <0.001), TTP ≥4 s (*r* = 0.70, *p* <0.001), TTP ≥6 s (*r* = 0.65, *p* <0.001), T_max_ ≥4 s (*r* = 0.73, *p* <0.001), T_max_ ≥6 s (*r* = 0.71, *p* <0.001), T_max_ ≥8 s (*r* = 0.66, *p* <0.001) and T_max_ ≥10s (*r* = 0.53, *p* = 0.002). Using the dichotomized thresholds, patients with high volumes of TTP ≥6 s and T_max_ ≥8 s had a higher median NIHSS score at discharge as those with low volumes (median NIHSS score for both parameters: 4 IQR: (2;13) vs. 1 (IQR 0;3), respectively, *p* = 0.009).

## Discussion

In the current study, we showed that i) automatic measurement of TTP and T_max_ volume with standardized thresholds predicts short term outcome in patients with unilateral acute sICA stenosis, ii) the visually assessed TTP volume did not differentiate between patients with favorable and unfavorable outcome, iii) the degree of stenosis is not different between patients with favorable and unfavorable outcome.

i) Both the TTP (≥4 and ≥6 s) and the T_max_ volumes discriminated between patients with favorable and unfavorable outcome in univariate analysis. The predictive value of these parameters was independent of the clinical stroke severity (NIHSS score on admission) or the stroke size (DWI lesion volume) in multivariate analysis. We hypothesize that the reason for the prediction of short term outcome by perfusion parameters could be infarct growth after the initial MRI and before recanalization. In the DEFUSE 2 trial, infarct growth was increased in patients with severe hypoperfusion [18]. In addition, further embolic strokes might have occurred during the hospital stay. It has been reported that the impaired clearance of emboli might be one of the causes for the increased stroke risk with hemodynamically relevant sICA stenosis [19], which is also supposed to be the link to the increased risk of stroke in patients with impaired cerebrovascular reserve [20]. Unfortunately, follow up MRI were not available for our patients to investigate these interpretations. We speculate that DWI lesion volume was not an independent predictor because of the pathophysiological characteristics of patients with ICA stenosis. In severe ICA stenosis patients with only small embolic DWI lesions and a large hypoperfused area can be found. In addition, embolic DWI lesions can occur in one area of the brain while a hemodynamic hypoperfusion is found in another area (Additional file 3: Figure S2). In our sample, NIHSS score on admission was therefore the superior predictor of outcome compared to DWI lesion volume.
  Another finding in our study was that the thresholds used to dichotomize the T_max_ and TTP volumes were relatively low. This is of relevance as it can be assumed that for example in patients with additional middle cerebral artery occlusion, any relevant pathological volume with these thresholds will have an impact on outcome. Whether these thresholds can be used for therapeutic decisions has to be evaluated in further trials.
ii) The visual assessment (eyeballing) of TTP maps derived from the MR-scanner software is widely used for clinical decision making. In the present analysis, this method does not predict outcome and can hence misguide therapeutic decisions. It was reported before that visually assessed TTP abnormalities do not correlate with relevant hypoperfusion [21]. We assume that the TTP maps with threshold performed better compared to the visually assessed TTP maps from the scanner software as they were normalized using TTP values of the white matter of the unaffected hemisphere contralateral to the stenosis. Based on our results, we suspect that irrelevant TTP increases occur mainly in the interval of 0–4 s as an elevated volume of TTP ≤4 s did not differentiate patients with favorable and unfavorable outcome. Our findings go in hand with a study reporting clinically irrelevant increases of TTP in patients with acute sICA stenosis [7]. Similar results have been obtained in other studies [8, 22]. However, in contrast to our study, the patient population in these studies was highly heterogeneous and included patients with intracranial stenosis and occlusions. The heterogeneity in this collective could potentially have changed the perfusion profiles dramatically.
iii) In our study we could show that the short term outcome in patients with ICA stenosis and hypoperfusion is not dependent on the degree of stenosis. A patient with a 70 % stenosis can have more hypoperfusion due to less collateralization and therefore a worse outcome compared to a patient with a 90 % stenosis. This is supported by our TCD findings that did not show a correlation to outcome either. MR perfusion may help to assess whether hypoperfusion caused by ICA stenosis is clinically relevant and whether a patient might benefit from emergency revascularization.

Our finding that CAS and TEA did not have an effect on short term outcome in multivariate analysis is not surprising as they are performed to reduce the long term risk of recurrent ischemia. The significant difference in univariate analysis might be a biased result. Altogether, four patients were not treated with CAS or TEA. One of them was a female patient with a 50 % stenosis who does not have a strong indication for recanalization according to the German national guidelines (http://www.awmf.org/leitlinien/detail/ll/004-028.html). In the other three patients, recanalization was recommended but refused by the patients or their relatives due to the poor clinical condition of the patients. We therefore assume that a part of the significant influence of therapy in the univariate analysis must be attributed to the fact that patients who refused recanalization might cause a skewed distribution.

Whether the degree of hypoperfusion can also help to predict the risk of recurrent ischemia is unclear. It has been shown that other factors than the degree of stenosis can influence the effect on outcome in patients undergoing CEA [23]. Currently, a randomized trial is examining the effect of CEA on patients with carotid artery stenosis that were stratified by a carotid artery risk score (European Carotid Surgery Trial II, www.ecst-2.com, ISRCTN 97744893). Our findings might have clinical implications in these patients if data from perfusion weighted imaging were added to a risk-prediction-score which can be used for therapeutic decisions.

Further results of our study in univariate analysis showed, that patients with diabetes more frequently had an unfavorable outcome, which could not be confirmed in the multivariate analysis. It might have been a significant predictor in a larger population as other studies have already shown the influence of blood glucose level on outcome [24, 25].

Limitations of the current study are mainly caused by its retrospective design. Patients with favorable and unfavorable outcome had different baseline NIHSS score. We controlled for this difference by including it in the multivariate logistic regression. Another difference stands out in the time until the MRI was performed. 26 % in the group with favorable outcome compared to 11 % in the group with unfavorable outcome received their MRI after 72 h. Although we cannot exclude a bias, the difference did not reach statistical significance in univariate analysis. Regarding the clinical outcome, there is an imbalance of patients (23 vs. 9), which could have led to distortions of the statistical analysis. Another limitation is the heterogeneity of the patient cohort that was increased because the presenting event could be either TIA or stroke. Furthermore, we exclusively used mRS at discharge as outcome measures, while especially for therapeutic decisions the long-term risk of stenosis might be more important. Additionally, the definition of thresholds and applying these thresholds in the same patient cohort may lead to a distortion of the effect, which therefore has to be replicated. It was not possible to split up the patients in a derivation and validation cohort because the number of patients was too small. Although it is likely that ICA stenosis caused the stroke in our patients, other etiologies cannot be excluded which could lead to a misinterpretation of the results.

## Conclusions

In conclusion, our study indicates that automatically calculated volumes T_max_ ≥8 s and TTP ≥6 s can stratify patients according to their outcome, while the frequently used visual assessment of TTP maps cannot. Future prospective studies with larger patient number should investigate if the proposed differentiation of patients based on T_max_ maps can help to stratify patients for recanalization therapy.

### Ethical approval and consent to participate

The study was approved by the ethics committee of the University of Heidelberg, Germany (statement S-330/2012). Due to the retrospective nature of this study the ethics committee did not require subsequent informed written consent of the included patients.

### Availability of data and material

All relevant data are included in the manuscript. The authors have no additional data to share.

## Additional files

Additional file 1: Figure S1. CT Angiography of the patient shown in Fig. 2. (TIF 1426 kb) Additional file 2: Table S1. Parameters of the ROC-Curve-Analysis. (DOCX 16 kb) Additional file 3: Figure S2. DWI lesions and T_max_ map of a patient with 90 % sICA stenosis. (TIF 3449 kb)

## Abbreviations

AUC: area under the curve
CAS: carotid artery stenting
CI: confidence interval
CT: computed tomography
DWI: diffusion weighted imaging
IQR: interquartile range
MCA: middle cerebral artery
MRI: magnetic resonance imaging
mRS: modified Rankin Scale
NASCET: North American Symptomatic Carotid Endarterectomy Trial
NIHSS: National Institute of Health Stroke Scale Score
OR: odds ratio
PWI: perfusion weighted imaging
ROC: receiver operating characteristic
sICA stenosis: symptomatic internal carotid artery stenosis
TCD: transcranial doppler sonography
TEA: thromboendarterectomy
T_max_: time to the maximum of the residue curve
TTP: time to the maximum of the tissue concentration time curve

## References

[1] Schulz UG, Rothwell PM (2003). Differences in vascular risk factors between etiological subtypes of ischemic stroke: importance of population-based studies. Stroke.

[2] North-American-Symptomatic-Carotid-Endarterectomy-Trial-Collaborators (1991). Beneficial effect of carotid endarterectomy in symptomatic patients with high-grade carotid stenosis. N Engl J Med.

[3] Ringleb PA, Allenberg J, Bruckmann H, Eckstein HH, Fraedrich G, Hartmann M, Hennerici M, Jansen O, Klein G, Kunze A (2006). 30 day results from the SPACE trial of stent-protected angioplasty versus carotid endarterectomy in symptomatic patients: a randomised non-inferiority trial. Lancet.

[4] Arning C, Widder B, von Reutern GM, Stiegler H, Gortler M (2010). [Revision of DEGUM ultrasound criteria for grading internal carotid artery stenoses and transfer to NASCET measurement]. Ultraschall Med.

[5] Momjian-Mayor I, Baron JC (2005). The pathophysiology of watershed infarction in internal carotid artery disease: review of cerebral perfusion studies. Stroke.

[6] Nasel C, Azizi A, Wilfort A, Mallek R, Schindler E (2001). Measurement of time-to-peak parameter by use of a new standardization method in patients with stenotic or occlusive disease of the carotid artery. AJNR Am J Neuroradiol.

[7] Neumann-Haefelin T, Wittsack HJ, Fink GR, Wenserski F, Li TQ, Seitz RJ, Siebler M, Modder U, Freund HJ (2000). Diffusion- and perfusion-weighted MRI: influence of severe carotid artery stenosis on the DWI/PWI mismatch in acute stroke. Stroke.

[8] Zaro-Weber O, Moeller-Hartmann W, Heiss WD, Sobesky J (2010). Maps of time to maximum and time to peak for mismatch definition in clinical stroke studies validated with positron emission tomography. Stroke.

[9] Calamante F, Christensen S, Desmond PM, Ostergaard L, Davis SM, Connelly A (2010). The physiological significance of the time-to-maximum (Tmax) parameter in perfusion MRI. Stroke.

[10] Olivot JM, Mlynash M, Thijs VN, Kemp S, Lansberg MG, Wechsler L, Bammer R, Marks MP, Albers GW (2009). Optimal Tmax threshold for predicting penumbral tissue in acute stroke. Stroke.

[11] Davis SM, Donnan GA, Parsons MW, Levi C, Butcher KS, Peeters A, Barber PA, Bladin C, De Silva DA, Byrnes G (2008). Effects of alteplase beyond 3 h after stroke in the Echoplanar Imaging Thrombolytic Evaluation Trial (EPITHET): a placebo-controlled randomised trial. Lancet Neurol.

[12] Broderick JP, Palesch YY, Demchuk AM, Yeatts SD, Khatri P, Hill MD, Jauch EC, Jovin TG, Yan B, Silver FL (2013). Endovascular therapy after intravenous t-PA versus t-PA alone for stroke. N Engl J Med.

[13] Yushkevich PA, Piven J, Hazlett HC, Smith RG, Ho S, Gee JC, Gerig G (2006). User-guided 3D active contour segmentation of anatomical structures: significantly improved efficiency and reliability. NeuroImage.

[14] Mouridsen K, Christensen S, Gyldensted L, Ostergaard L (2006). Automatic selection of arterial input function using cluster analysis. Magn Reson Med.

[15] Sobesky J, Zaro Weber O, Lehnhardt FG, Hesselmann V, Thiel A, Dohmen C, Jacobs A, Neveling M, Heiss WD (2004). Which time-to-peak threshold best identifies penumbral flow? A comparison of perfusion-weighted magnetic resonance imaging and positron emission tomography in acute ischemic stroke. Stroke.

[16] Lansberg MG, Lee J, Christensen S, Straka M, De Silva DA, Mlynash M, Campbell BC, Bammer R, Olivot JM, Desmond P (2011). RAPID automated patient selection for reperfusion therapy: a pooled analysis of the Echoplanar Imaging Thrombolytic Evaluation Trial (EPITHET) and the Diffusion and Perfusion Imaging Evaluation for Understanding Stroke Evolution (DEFUSE) Study. Stroke.

[17] Rochon JGM, Kieser M (2012). To test or not to test: preliminary assessment of normality when comparing two independent samples. BMC Med Res Methodol.

[18] Olivot JM, Mlynash M, Inoue M, Marks MP, Wheeler HM, Kemp S, Straka M, Zaharchuk G, Bammer R, Lansberg MG (2014). Hypoperfusion intensity ratio predicts infarct progression and functional outcome in the DEFUSE 2 Cohort. Stroke.

[19] Caplan LR, Hennerici M (1998). Impaired clearance of emboli (washout) is an important link between hypoperfusion, embolism, and ischemic stroke. Arch Neurol.

[20] Gupta A, Chazen JL, Hartman M, Delgado D, Anumula N, Shao H, Mazumdar M, Segal AZ, Kamel H, Leifer D (2012). Cerebrovascular reserve and stroke risk in patients with carotid stenosis or occlusion: a systematic review and meta-analysis. Stroke.

[21] Takasawa M, Jones PS, Guadagno JV, Christensen S, Fryer TD, Harding S, Gillard JH, Williams GB, Aigbirhio FI, Warburton EA (2008). How reliable is perfusion MR in acute stroke? Validation and determination of the penumbra threshold against quantitative PET. Stroke.

[22] Asdaghi N, Hill MD, Coulter JI, Butcher KS, Modi J, Qazi A, Goyal M, Demchuk AM, Coutts SB (2013). Perfusion MR predicts outcome in high-risk transient ischemic attack/minor stroke: a derivation-validation study. Stroke.

[23] Rothwell PM, Mehta Z, Howard SC, Gutnikov SA, Warlow CP (2005). Treating individuals 3: from subgroups to individuals: general principles and the example of carotid endarterectomy. Lancet.

[24] Ahmed N, Davalos A, Eriksson N, Ford GA, Glahn J, Hennerici M, Mikulik R, Kaste M, Lees KR, Lindsberg PJ (2010). Association of admission blood glucose and outcome in patients treated with intravenous thrombolysis: results from the Safe Implementation of Treatments in Stroke International Stroke Thrombolysis Register (SITS-ISTR). Arch Neurol.

[25] Bruno A, Biller J, Adams HP, Clarke WR, Woolson RF, Williams LS, Hansen MD (1999). Acute blood glucose level and outcome from ischemic stroke. Trial of ORG 10172 in Acute Stroke Treatment (TOAST) Investigators. Neurology.

