# Supplementary figures and images for: Time-dependent parameter of perfusion imaging as independent predictor of clinical outcome in symptomatic carotid artery stenosis
Source: BMC Neurol. 2016 Apr 19;16:50. doi: 10.1186/s12883-016-0576-5 (PMC4837540; doi:10.1186/s12883-016-0576-5)

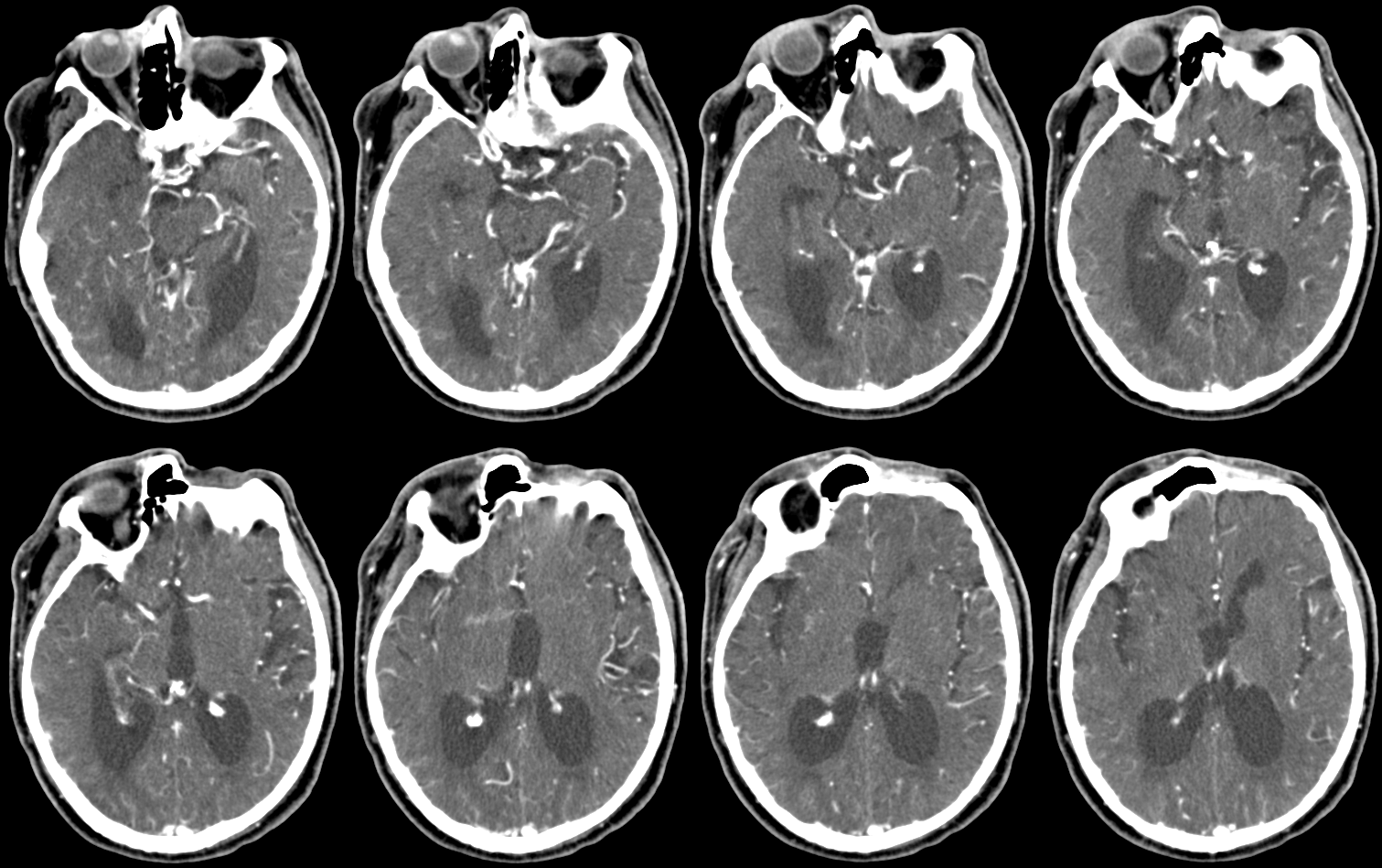

Supplement: Additional file 1: Figure S1. — CT Angiography of the patient shown in Fig. 2. (TIF 1426 kb) [file 12883_2016_576_MOESM1_ESM.tif]

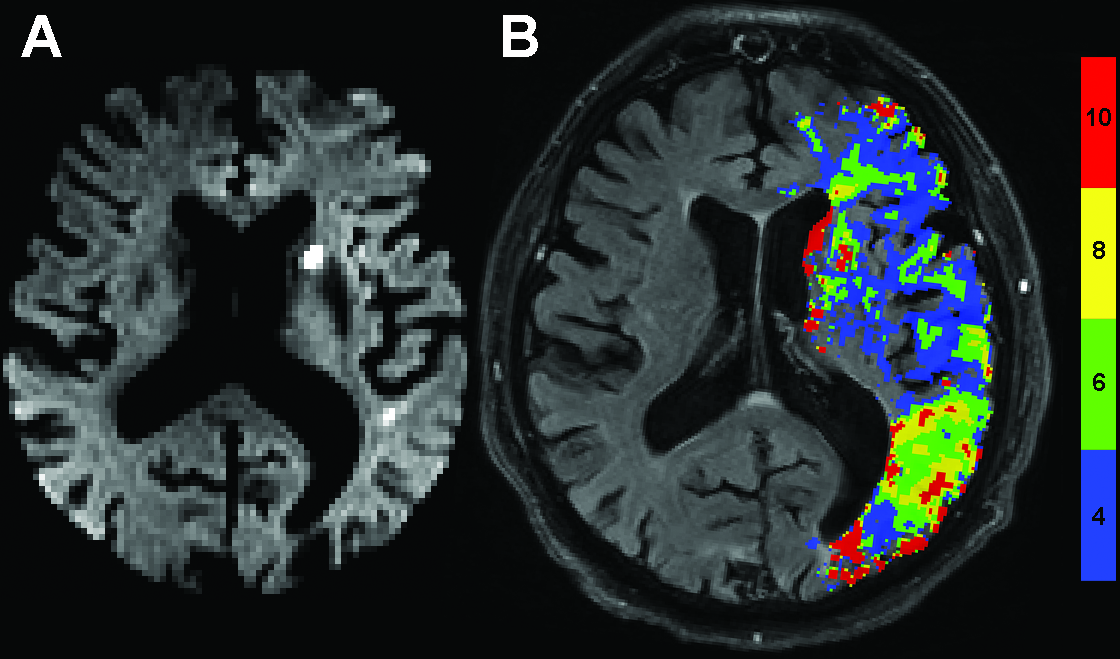

Supplement: Additional file 3: Figure S2. — DWI lesions and Tmax map of a patient with 90 % sICA stenosis. (TIF 3449 kb) [file 12883_2016_576_MOESM3_ESM.tif]
