# Supplementary material for: Time-dependent parameter of perfusion imaging as independent predictor of clinical outcome in symptomatic carotid artery stenosis
Source: BMC Neurol. 2016 Apr 19;16:50. doi: 10.1186/s12883-016-0576-5 (PMC4837540; doi:10.1186/s12883-016-0576-5)
Supplement: Additional file 2: Table S1. — Parameters of the ROC-Curve-Analysis. (DOCX 16 kb) [file 12883_2016_576_MOESM2_ESM.docx]

Supplementary Table:

**Tab.S1: Parameters of the ROC-Curve-Analysis**

| **Parameter** | **Area** | **Error** | **p-Value** | **95% Confidence-interval** | | **Sensitivity** | **Specificity** | **Threshold**  **Vol. (ml)** |
| --- | --- | --- | --- | --- | --- | --- | --- | --- |
| **T_max_ >4** | 0.734 | 0.112 | 0.042 | 0.515 | 0.954 | 0.778 | 0.565 | 2.198 |
| **T_max_ >6** | 0.773 | 0.106 | 0.018 | 0.566 | 0.980 | 0.778 | 0.783 | 1.281 |
| **T_max_ >8** | 0.787 | 0.105 | 0.013 | 0.581 | 0.994 | 0.778 | 0.826 | 0.942 |
| **T_max_ >10** | 0.768 | 0.105 | 0.020 | 0.562 | 0.975 | 0.778 | 0.826 | 0.741 |
| **TTP>2** | 0.686 | 0.113 | 0.107 | 0.465 | 0.906 | 0.778 | 0.391 | 5.176 |
| **TTP>4** | 0.773 | 0.099 | 0.018 | 0.578 | 0.968 | 0.778 | 0.652 | 2.214 |
| **TTP>6** | 0.836 | 0.092 | 0.004 | 0.656 | 1.000 | 0.778 | 0.870 | 1.423 |
| **TTP visual** | 0.560 | 0.126 | 0.600 | 0.313 | 0.808 | 0.667 | 0.565 | 85.750 |
